# Three-Dimensional Vibration Analysis of Laminated Composite Rectangular Plate with Cutouts

**DOI:** 10.3390/ma13143113

**Published:** 2020-07-13

**Authors:** Kai Xue, Wenhao Huang, Qiuhong Li

**Affiliations:** College of Mechanical and Electrical Engineering, Harbin Engineering University, Harbin 150001, China; xuekai@hrbeu.edu.cn (K.X.); liqiuhong@hrbeu.edu.cn (Q.L.)

**Keywords:** three-dimensional vibration, three-dimensional elasticity theory, laminated composite structures, plate with cutouts, Fourier series

## Abstract

The main purpose of this paper is to establish an analysis model of laminated composite rectangular plate with/without cutouts on the basis of three-dimensional elasticity theory and provide the exact three-dimensional solution. In the present work, the effect of the cutout is considered by subtracting the energies of cutouts from the total energy of the entire plate. The standard three-dimensional trigonometric cosine Fourier series and auxiliary Fourier series are chosen as admissible functions, and the Hamilton’s principle and Rayleigh-Ritz procedure are used to obtain the exact solution. In order to verify the effectiveness and reliability of the proposed method, some numerical results are obtained, and the results are compared with the ones available in the literature or finite element analysis. Finally, the effects of some key parameters which will affect the vibration characteristics are analyzed, and the non-dimensional frequency parameters are obtained, which can serve as benchmark data for the future research.

## 1. Introduction

A composite material is a heterogeneous material formed by combining two or more constituent materials with different properties integrated together to achieve enhanced structural performance. Owing to the high specific strength and stiffness, high fatigue resistance, light weight, and good thermal stability compared with conventional heterogeneous materials, applications of composite structures are employed in diverse fields that include vehicles, aircraft, turbines, architecture, submarines, and so on. [1,2]. With the continuous development of composite material manufacturing technology, composite materials have developed into one of the four major material systems in parallel with metal materials, polymer materials, inorganic non-metal materials, and laminated composite plates have become the basic structural components of most engineering structures. Therefore, it is necessary for engineers to have a thorough understanding of the vibration characteristics of laminated composite plates to ensure the reliability of structural performance.

In recent decades, the prediction of vibration characteristics of composite structure has attracted great interest from engineers and experts, and significant research advances have been achieved. Furthermore, a large amount of analytical theories have been established, such as the classical laminated plate theory (CLPT), first-order shear deformation theory (FSDT), higher-order shear deformation theory (HSDT), zigzag theory (ZZT), and three-dimensional elasticity theory (3DT).

The classical laminated plate theory is the simplest theory of all plate theories, Love [3] proposed this theory which is based on the Kirchhoff’s hypothesis in the late 19th century. According to the theory, the displacement field is expressed as three displacement components along the *x*, *y*, *z* coordinate directions of a point on the midplane, thus the three-dimensional structures are reduced to two-dimensional structures. The simple form of displacement fields and reduction of variables result in simple equations and high calculation efficiency, it is applicable to the thin multi-layer composite structures. By neglecting the influence of the transverse shear deformation and rotational inertia, the vibration characteristics of the rather thick plates obtained by this theory are inaccurate, which will lead to smaller displacement and stress and higher natural frequency of the plates. Even for thin laminated composite plates, when the Young’s modulus of the material is small, the transverse shear deformation cannot be neglected, and the resultant deviation of the theory increases sharply. CLPT remains a popular approach to solving the vibration problems of thin structures, and many research groups have proposed some theories in order to achieve better results based on CLPT. Gorman and Ding [4,5] exploited the superposition method and Galerkin method to obtain the exact analytical solution of laminated composite plates under free boundary conditions. Nallim et al. [6,7,8] used the Ritz method in conjunction with natural coordinates to express the geometry of general plates by using a set of beam characteristic orthogonal polynomials. Vibration characteristics of general quadrilateral plates, trapezoidal plates, skew plates, and rhomboidal plates with angle-ply layers have been studied. Secgin et al. [9] proposed a discrete singular convolution (DSC) approach by using a grid discretization based on distribution theory and wavelets. Cosentino and Weaver [10] developed a mixed theory by means of Reissner’s variational approach based on Castigliano’s principle of least work in conjunction with a Lagrange multiplier method to assess the effect of transverse shear stresses of thick composite laminates and sandwich plates.

In order to obtain more accurate vibration characteristics of laminated composite structures, Reissner [11] and Mindlin [12] proposed the first-order shear deformation theory by including the transverse shear deformation, and assumed that the shear force and strain are constant in the thickness coordinate direction, which is not consistent with the parabola distribution of shear force and strain. Therefore, the shear correction factor has been introduced into this theory to correct the shear strain and stress [13,14,15], which is related to the material, geometry, loads, boundary conditions, and laminations. The results obtained by the first-order shear deformation theory largely depend on the shear correction factor which is generally selected as 5/6 or $\pi^{2}/12$. Subsequently, the higher-order shear deformation theory was proposed by Reddy [16,17] to overcome the shortcomings of CLPT or FSDT. Liew et al. [18] adopted the first-order shear deformation theory in the moving least squares differential quadrature procedure by using the moving least squares shape functions and their partial derivatives to obtain the weighting coefficients to predict the free vibration behavior of moderately thick symmetrically laminated composite plates. Karami et al. [19] applied the differential quadrature method (DQM) for free vibration analysis of moderately thick composite plates with edges elastically restrained against translation and rotation. The governing differential equations with their boundary conditions are transformed into algebraic equations by using differential quadrature rules in order to establish the eigenvalue equations, and the results showed that the DQM can yield an accurate solution using few grid points. Ngo-Cong et al. [20] employed one-dimensional integrated RBF networks instead of conventional differentiated RBF networks to approximate the field variables, and the rectangular or non-rectangular plates are discretized by means of Cartesian grids. Carrera [21] reduced the third-order model from five displacement variables to three by imposing homogeneous stress conditions with correspondence to the plate top-surface, and then modified it to apply to non-homogeneous stress conditions. Closed form solution has been obtained for both stresses and displacements in the case of harmonic loadings and simply supported boundary conditions. Chen et al. [22] employed the p-Ritz method by using the polynomials as the admissible trial displacement and rotation functions to study the vibration of laminated plates. Aydogdu [23] chose different types of shape functions to determine the distribution of the transverse shear strains and stresses along the thickness according to 3-D results to present a new higher order shear deformation theory. The new shear model was used to analyze bending, free vibration, and bulking of laminated composite plates. Ferreira et al. [24] applied a multi-quadrics radial basis function method and a third-order shear deformation theory to solve the analysis of isotropic and symmetric laminated composite thick beams and plates. Liu et al. [25] used the radial basis functions with polynomial reproduction to present the problem domain by a set of scattered nodes in its support domain. The static deflection, free vibration, and bulking analysis of laminated composite plates were presented.

Generally, the solution of higher-order shear deformation theory is more accurate than that obtained by using the first-order shear deformation theory due to the shear strain being distributed as a cubic function in the thickness direction; however, they introduce rather sophisticated formulations and boundary terms that are not easily applicable or yet understood [26]. All of the above methods belong to the equivalent two-dimensional methods, and the two-dimensional methods cannot satisfy the piecewise continuous displacement requirement and insufficient considerations for the transverse stress fields between layers. Murakami [27] developed the zigzag theory by including a zigzag shaped function to approximate the thickness variation of in-plane displacement. Sciuva [28,29] proposed a piecewise linear zigzag theory and then a non-linear third-order zigzag theory which accounts for continuous inter-laminar transverse shearing stresses at the interfaces between any two adjacent layers. Kapuria et al. [30,31,32,33] applied a new zigzag theory to analyze the static, dynamic, bulking, and thermal problems of laminated composite beams or plates. Pandit et al. [34] proposed an improved higher order zigzag theory for the static analysis of laminated sandwich plates with soft compressible core. The variation of in-plane displacements is assumed to be cubic for both the face sheets and the core, the transverse displacement is assumed to vary quadratically within the core while it remains constant through the faces. Khandelwal et al. [35] developed an improved FE plate model based on refined higher order shear deformation theory (RHSDT) and a least square error method (LSE) to accurately predict the deflections and stresses of composite and sandwich laminates. The $C^{1}$ zigzag theory was proposed based on the nine node $C^{0}$ element which satisfies the inter-laminar shear stress continuity conditions at the layer interfaces and zero transverse shear stress conditions at the top and bottom of the plate. The development of zigzag theory can be found in [36]. Generally, there are many variables in the zigzag theory and the displacement field functions are relatively complicated.

The three-dimensional elasticity theory does not rely on any hypothesis, each sublayer of the structure is regarded as a three-dimensional entity which is described as an independent function, and it satisfies the continuity of interlaminar displacement and stress requirement. Liew et al. [37] presented a continuum three-dimensional Ritz formulation by selecting sets of orthogonally generated polynomial functions as shape functions. The frequency parameters and mode shapes of thick plates have been solved with five practical groups of boundary conditions. Senthil et al. [38] presented a three-dimensional exact analytical solution and benchmark results for the free and forced vibrations of simply supported functionally graded rectangular plates by using suitable displacement functions in order to reduce the governing partial differential equations to a set of coupled ordinary differential equations in the thickness direction, which are solved by the power series method. Zhou et al. [39,40,41,42,43] used Chebyshev polynomials and static beam functions as admissible functions in conjunction with Rayleigh-Ritz method to study the three-dimensional vibrations of rectangular, skew, elliptical, and circular annular plates, as well as solid and hollow circular cylinders with different boundary conditions. Qu et al. [44] employed a multilevel partitioning hierarchy, viz., multilayered parallelepiped, individual layer and layer segment, which are based on the exact three-dimensional elasticity theory to analyze the free and transverse vibrations of multilayered laminated composite and sandwich beams, plates, and solids with various boundary conditions. The displacement components of each layer segment are approximated as the product of orthogonal polynomials and/or trigonometric functions.

The structure with cutouts is common in the practical engineering, the cutouts are used to reduce the weight or provide operational and maintenance access. The existence of cutouts changes the dynamic characteristics of the structure and damages the service life of the structures. It is necessary to study the effect of the cutouts on the vibration response of the structures. Liew et al. [45] employed a domain decomposition method by using a basic L-shaped element which is divided into appropriate sub-domains that are dependent upon the location of the cutouts as the basic element to solve the free vibration of plates with central cutouts. Kumar et al. [46] developed a finite element formulation based on higher-order shear deformation theory and Hamilton’s principle to study the vibration response of thick square composite plates with a central rectangular cutout. The effect of material orthotropy, boundary conditions, side-to-thickness ratio, delamination size, and location around the cutout is investigated. Sakiyama et al. [47] proposed an approximate method and transformed the problem into an equivalent square plate with non-uniform thickness by considering the cutout as an extremely thin part of the equivalent plate. The Green function is used to obtain the discrete solution and characteristic equation of the plate. Laura et al. [48,49,50,51] have made some achievements in the vibration characteristics of plates with cutouts by applying the Rayleigh-Ritz variational method. Shufrin et al. [52] presented a new semi-analytical variational extended the Kantorovich method to model rectangular plates with variable thickness and cutouts. The plate thickness and deflections are represented as a finite sum of multiplications of one-dimensional functions. Kwak et al. [53] developed an independent coordinated coupling method (ICCM) in which the energies of the plate domain and the cutout domain are derived independently and the two independent coordinates are coupled by imposing kinematic relations. The vibration analysis of a rectangular plate with a rectangular cutout or a circular cutout is investigated. Huang [54,55] then applied this method to solve the problem of orthotropic composite laminated plate and functionally graded carbon nanotube-reinforced plate. The existing literature surveys show that most of the studies on the vibration of rectangular plates with cutouts are based on the traditional two-dimensional theories, and that three-dimensional exact solutions are very rare.

In this paper, a unified analysis model of the vibration characteristics of the laminated composite plates with/without cutouts is established, and the three-dimensional exact solution is provided with different boundary conditions. According to the energy principle, the Lagrangian energy functions of the plate and the cutout are obtained, and then a standard three-dimensional trigonometric cosine function and auxiliary Fourier series are selected as the admissible functions. The basic idea of solving the free vibration of a plate with a cutout is to subtract the energy of the cutout part from the total energy of the plate. The governing equations are solved by using the Hamilton’s principle and Rayleigh-Ritz method. In order to verify the reliability of the method, some numerical examples are carried out to compare with those available in the literature and the finite element analysis. Furthermore, the effect of some key parameters which may affect the vibration characteristics is conducted, including the position of the cutouts, the size of the cutouts, the laminations, the boundary conditions, and the layer fiber direction angle. The non-dimensional frequency parameters are shown which can serve as a benchmark solution for future research.

## 2. Theoretical Formulations

### 2.1. Description of the Model

**Figure 1:**
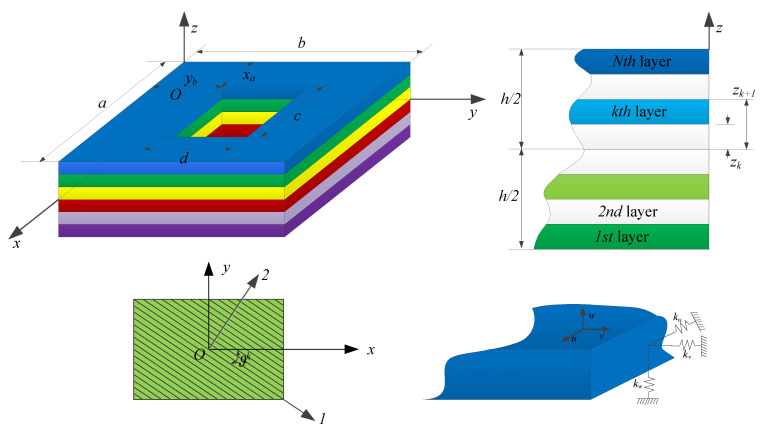
Model of laminated composite rectangular plate with a rectangular cutout.

Consider a multilayered laminated composite rectangular plate with a rectangular cutout, as shown in Figure 1. The global coordinate system (*x*, *y*, *z*) which located in the middle surface of the structure is considered to present the dimensions. The length, width, and thickness of the plate are assumed as *a*, *b*, and *h*, the dimensions of the rectangular cutout in the *x* and *y* directions are *c* and *d*, the distances of the cutout in the *x* and *y* directions with respect to the coordinated point *O* are *x_a_* and *y_b_*, the coordinates of the cutout center are *x_h_* and *y_h_*, whereas *x_h_* = *x_a_* + *c/2* and *y_h_* = *y_b_* + *d/2*, respectively. The middle surface displacements of the plate in the *x*, *y*, and *z* directions are denoted by *u*, *v*, and *w*, respectively. The number of the layers of the laminated composite plate is assumed to be *N*, and the *kth* layer has a thickness of *h_k_*, where the *h_k_ = z_k+_*_1_ *− z_k_*, and the *z_k+_*_1_ and *z_k_* are the distances from the top surface and the bottom surface of the layer to the referenced middle surface. The principal coordinates of the laminated composite plate in the *k*th layer are denoted as 1, 2, and 3, the angle between the material axis and x coordinate is denoted by $\vartheta^{k}$. The artificial springs are introduced in order to simulate different kinds of boundary conditions, three groups of springs *k_u_*, *k_v_*, and *k_w_*, which are distributed uniformly along the edges. The stiffness of the springs can take any value from zero to infinity to model the classical boundary conditions or elastic boundary conditions.

### 2.2. Stress-Strain Relations and Stress Resultants

Based on the three-dimensional elasticity theory and small deformation theory, the strain—displacement relations of the *k*th layer for the plate are of the form

(1) $$\varepsilon_{x}^{k}=\frac{\partial u^{k}}{\partial x}$$

(2) $$\varepsilon_{y}^{k}=\frac{\partial v^{k}}{\partial y}$$

(3) $$\varepsilon_{z}^{k}=\frac{\partial w^{k}}{\partial z}$$

(4) $$\gamma_{yz}^{k}=\frac{\partial w^{k}}{\partial y}+\frac{\partial v^{k}}{\partial z}$$

(5) $$\gamma_{xz}^{k}=\frac{\partial u^{k}}{\partial z}+\frac{\partial w^{k}}{\partial x}$$

(6) $$\gamma_{xy}^{k}=\frac{\partial u^{k}}{\partial y}+\frac{\partial v^{k}}{\partial x}$$

where the *u^k^*, *v^k^*, and *w^k^* are the middle surface displacement components of an arbitrary point of the plate in the *x*, *y*, and *z* directions, respectively. $\varepsilon_{x}^{k}$, $\varepsilon_{y}^{k}$, $\varepsilon_{z}^{k}$, $\gamma_{yz}^{k}$, $\gamma_{xz}^{k}$, and $\gamma_{xy}^{k}$ are the normal and shear strain components in the *x*, *y*, and *z* coordinate system.

According to the generalized Hooke’s law, the corresponding stress-strain relations in the *k*th layer of the plate are obtained as

(7) $$\left[\begin{array}{c} \sigma_{x}^{k}\\ \sigma_{y}^{k}\\ \sigma_{z}^{k}\\ \tau_{yz}^{k}\\ \tau_{xz}^{k}\\ \tau_{xy}^{k} \end{array}\right]=\left[\begin{array}{cccccc} \bar{C_{11}^{k}} & \bar{C_{12}^{k}} & \bar{C_{13}^{k}} & 0 & 0 & \bar{C_{16}^{k}}\\ \bar{C_{12}^{k}} & \bar{C_{22}^{k}} & \bar{C_{23}^{k}} & 0 & 0 & \bar{C_{26}^{k}}\\ \bar{C_{13}^{k}} & \bar{C_{23}^{k}} & \bar{C_{33}^{k}} & 0 & 0 & \bar{C_{36}^{k}}\\ 0 & 0 & 0 & \bar{C_{44}^{k}} & \bar{C_{45}^{k}} & 0\\ 0 & 0 & 0 & \bar{C_{45}^{k}} & \bar{C_{55}^{k}} & 0\\ \bar{C_{16}^{k}} & \bar{C_{26}^{k}} & \bar{C_{36}^{k}} & 0 & 0 & \bar{C_{66}^{k}} \end{array}\right]\left[\begin{array}{c} \varepsilon_{x}^{k}\\ \varepsilon_{y}^{k}\\ \varepsilon_{z}^{k}\\ \gamma_{yz}^{k}\\ \gamma_{xz}^{k}\\ \gamma_{xy}^{k} \end{array}\right]$$

where the $\sigma_{x}^{k}$, $\sigma_{y}^{k}$, $\sigma_{z}^{k}$, $\tau_{yz}^{k}$, $\tau_{xz}^{k}$, and $\tau_{xy}^{k}$ are the normal and shear stress components of the *k*th layer of the plate. The constants $\bar{C_{ij}^{k}}(i,j=1,2,\cdots ,6)$ are elastic stiffness coefficients of the layer, which can be expressed as

(8) $$\bar{C_{11}^{k}}=C_{11}^{k}\cos^{4}\vartheta^{k}+(2C_{12}^{k}+4C_{66}^{k})\cos^{2}\vartheta^{k}\sin^{2}\vartheta^{k}+C_{22}^{k}\sin^{4}\vartheta^{k}$$

(9) $$\bar{C_{12}^{k}}=(C_{11}^{k}+C_{22}^{k}-4C_{66}^{k})\cos^{2}\vartheta^{k}\sin^{2}\vartheta^{k}+C_{12}^{k}(\cos^{4}\vartheta^{k}+\sin^{4}\vartheta^{k})$$

(10) $$\bar{C_{13}^{k}}=C_{13}^{k}\cos^{2}\vartheta^{k}+C_{23}^{k}\sin^{2}\vartheta^{k}$$

(11) $$\bar{C_{16}^{k}}=(C_{11}^{k}-C_{22}^{k}-2C_{66}^{k})\cos^{3}\vartheta^{k}\sin\vartheta^{k}+(C_{12}^{k}-C_{22}^{k}+2C_{66}^{k})\cos\vartheta^{k}\sin^{3}\vartheta^{k}$$

(12) $$\bar{C_{22}^{k}}=C_{11}^{k}\sin^{4}\vartheta^{k}+(2C_{12}^{k}+4C_{66}^{k})\cos^{2}\vartheta^{k}\sin^{2}\vartheta^{k}+C_{22}^{k}\cos^{4}\vartheta^{k}$$

(13) $$\bar{C_{23}^{k}}=C_{23}^{k}\cos^{2}\vartheta^{k}+C_{13}^{k}\sin^{2}\vartheta^{k}$$

(14) $$\bar{C_{26}^{k}}=(C_{11}^{k}-C_{12}^{k}-2C_{66}^{k})\cos\vartheta^{k}\sin^{3}\vartheta^{k}+(C_{12}^{k}-C_{22}^{k}+2C_{66}^{k})\cos^{3}\vartheta^{k}\sin\vartheta^{k}$$

(15) $$\bar{C_{33}^{k}}=C_{33}^{k}$$

(16) $$\bar{C_{36}^{k}}=(C_{13}^{k}-C_{23}^{k})\cos\vartheta^{k}\sin\vartheta^{k}$$

(17) $$\bar{C_{44}^{k}}=C_{44}^{k}\cos^{2}\vartheta^{k}+C_{55}^{k}\sin^{2}\vartheta^{k}$$

(18) $$\bar{C_{45}^{k}}=(C_{55}^{k}-C_{44}^{k})\cos\vartheta^{k}\sin\vartheta^{k}$$

(19) $$\bar{C_{55}^{k}}=C_{55}^{k}\cos^{2}\vartheta^{k}+C_{44}^{k}\sin^{2}\vartheta^{k}$$

(20) $$\bar{C_{66}^{k}}=C_{66}^{k}(\cos^{4}\vartheta^{k}+\sin^{4}\vartheta^{k})+(C_{11}^{k}-2C_{12}^{k}+C_{22}^{k}-2C_{66}^{k})\cos^{2}\vartheta^{k}\sin^{2}\vartheta^{k}$$

where $C_{ij}^{k}(i,j=1,2,\cdots ,6)$ are material constants in the principal coordinate system (1, 2, and 3), which are expressed as

(21) $$C_{11}^{k}=\frac{1-\mu_{23}^{k}\mu_{32}^{k}}{E_{2}^{k}E_{3}^{k}\Delta }$$

(22) $$C_{12}^{k}=\frac{\mu_{21}^{k}+\mu_{23}^{k}\mu_{31}^{k}}{E_{2}^{k}E_{3}^{k}\Delta }=\frac{\mu_{12}^{k}+\mu_{32}^{k}\mu_{13}^{k}}{E_{1}^{k}E_{3}^{k}\Delta }$$

(23) $$C_{13}^{k}=\frac{\mu_{31}^{k}+\mu_{21}^{k}\mu_{32}^{k}}{E_{2}^{k}E_{3}^{k}\Delta }=\frac{\mu_{13}^{k}+\mu_{12}^{k}\mu_{23}^{k}}{E_{1}^{k}E_{2}^{k}\Delta }$$

(24) $$C_{22}^{k}=\frac{1-\mu_{13}^{k}\mu_{31}^{k}}{E_{1}^{k}E_{3}^{k}\Delta }$$

(25) $$C_{23}^{k}=\frac{\mu_{32}^{k}+\mu_{12}^{k}\mu_{31}^{k}}{E_{1}^{k}E_{3}^{k}\Delta }=\frac{\mu_{23}^{k}+\mu_{21}^{k}\mu_{13}^{k}}{E_{1}^{k}E_{2}^{k}\Delta }$$

(26) $$C_{33}^{k}=\frac{1-\mu_{12}^{k}\mu_{21}^{k}}{E_{1}^{k}E_{2}^{k}\Delta }$$

(27) $$C_{44}^{k}=G_{23}^{k}$$

(28) $$C_{55}^{k}=G_{13}^{k}$$

(29) $$C_{66}^{k}=G_{12}^{k}$$

(30) $$\Delta =\frac{1-\mu_{12}^{k}\mu_{21}^{k}-\mu_{23}^{k}\mu_{32}^{k}-\mu_{13}^{k}\mu_{31}^{k}-2\mu_{21}^{k}\mu_{32}^{k}\mu_{13}^{k}}{E_{1}^{k}E_{2}^{k}E_{3}^{k}}$$

where $E_{1}^{k}$,$E_{2}^{k}$, and $E_{3}^{k}$ are moduli of elasticity of the *k*th layer in the principle directions, $G_{12}^{k}$, $G_{13}^{k}$, and $G_{23}^{k}$ are shear moduli and $\mu_{ij}^{k}(i,j=1,2,3,\;i\neq j)$ are Poisson’s ratios. According to the symmetry of coefficients in the stress-strain relationship, the constants satisfy the following

(31) $$\frac{\mu_{ij}^{k}}{E_{i}^{k}}=\frac{\mu_{ji}^{k}}{E_{j}^{k}}(i,j=1,2,3)$$

An isotropic model can be obtained by letting: $E_{1}^{k}=E_{2}^{k}=E_{3}^{k}=E$, $\mu_{12}^{k}=\mu_{13}^{k}=\mu_{23}^{k}=\mu$, and $G_{12}^{k}=G_{13}^{k}=G_{23}^{k}=E/(2+2\mu )$, where E and μ are elastic modulus and Poisson’s ratio of the isotropic material, respectively.

### 2.3. Energy Functions

As shown in Figure 1, according to the three-dimensional elasticity theory, the energy functions of the plate and the cutouts are established independently in the global coordinate system, which can be expressed as

(32) $$T_{p}=\frac{1}{2}\int_{-h./2}^{h/2}\int_{0}^{b}\int_{0}^{a}[{\left(\frac{\partial u}{\partial t}\right)}^{2}+{\left(\frac{\partial v}{\partial t}\right)}^{2}+{\left(\frac{\partial w}{\partial t}\right)}^{2}]dxdydz$$

(33) $$T_{h}=\frac{1}{2}\int_{-h./2}^{h/2}\int_{y_{b}}^{y_{b}+d}\int_{x_{a}}^{x_{a}+c}[{\left(\frac{\partial u}{\partial t}\right)}^{2}+{\left(\frac{\partial v}{\partial t}\right)}^{2}+{\left(\frac{\partial w}{\partial t}\right)}^{2}]dxdydz$$

(34) $$\begin{array}{ll} U_{p} & =\frac{1}{2}{∭}_{V}\left[\sigma_{x}^{}\varepsilon_{x}^{}+\sigma_{y}^{}\varepsilon_{y}^{}+\sigma_{z}^{}\varepsilon_{z}^{}+\tau_{yz}^{}\gamma_{yz}^{}+\tau_{xz}^{}\gamma_{xz}^{}+\tau_{xy}^{}\gamma_{xy}^{}\right]dV\\ & =\frac{1}{2}\int_{0}^{h}\int_{0}^{b}\int_{0}^{a}\{\bar{C_{11}^{k}}{\left(\frac{\partial u}{\partial x}\right)}^{2}+\bar{C_{22}^{k}}{\left(\frac{\partial v}{\partial y}\right)}^{2}+\bar{C_{33}^{k}}{\left(\frac{\partial w}{\partial z}\right)}^{2}+\bar{C_{44}^{k}}{\left(\frac{\partial w}{\partial y}+\frac{\partial v}{\partial z}\right)}^{2}+\bar{C_{55}^{k}}{\left(\frac{\partial u}{\partial z}+\frac{\partial w}{\partial x}\right)}^{2}+\bar{C_{66}^{k}}{\left(\frac{\partial u}{\partial y}+\frac{\partial v}{\partial x}\right)}^{2}\\ & +2[\bar{C_{16}^{k}}\frac{\partial u}{\partial x}(\frac{\partial u}{\partial y}+\frac{\partial v}{\partial x})+\bar{C_{26}^{k}}\frac{\partial v}{\partial y}(\frac{\partial u}{\partial y}+\frac{\partial v}{\partial x})+\bar{C_{36}^{k}}\frac{\partial w}{\partial z}(\frac{\partial u}{\partial y}+\frac{\partial v}{\partial x})]\\ & +2(\bar{C_{12}^{k}}\frac{\partial u}{\partial x}\frac{\partial v}{\partial y}+\bar{C_{13}^{k}}\frac{\partial u}{\partial x}\frac{\partial w}{\partial z}+\bar{C_{23}^{k}}\frac{\partial v}{\partial y}\frac{\partial w}{\partial z})+2\bar{C_{45}^{k}}(\frac{\partial u}{\partial z}+\frac{\partial w}{\partial x})(\frac{\partial w}{\partial y}+\frac{\partial v}{\partial z})\}dxdydz \end{array}$$

(35) $$\begin{array}{ll} U_{h} & =\frac{1}{2}{∭}_{V}\left[\sigma_{x}^{}\varepsilon_{x}^{}+\sigma_{y}^{}\varepsilon_{y}^{}+\sigma_{z}^{}\varepsilon_{z}^{}+\tau_{yz}^{}\gamma_{yz}^{}+\tau_{xz}^{}\gamma_{xz}^{}+\tau_{xy}^{}\gamma_{xy}^{}\right]dV\\ & =\frac{1}{2}\int_{-h/2}^{h/2}\int_{y_{b}}^{y_{b}+d}\int_{x_{a}}^{x_{a}+c}\{\bar{C_{11}^{k}}{\left(\frac{\partial u}{\partial x}\right)}^{2}+\bar{C_{22}^{k}}{\left(\frac{\partial v}{\partial y}\right)}^{2}+\bar{C_{33}^{k}}{\left(\frac{\partial w}{\partial z}\right)}^{2}+\bar{C_{44}^{k}}{\left(\frac{\partial w}{\partial y}+\frac{\partial v}{\partial z}\right)}^{2}+\bar{C_{55}^{k}}{\left(\frac{\partial u}{\partial z}+\frac{\partial w}{\partial x}\right)}^{2}+\bar{C_{66}^{k}}{\left(\frac{\partial u}{\partial y}+\frac{\partial v}{\partial x}\right)}^{2}\\ & +2[\bar{C_{16}^{k}}\frac{\partial u}{\partial x}(\frac{\partial u}{\partial y}+\frac{\partial v}{\partial x})+\bar{C_{26}^{k}}\frac{\partial v}{\partial y}(\frac{\partial u}{\partial y}+\frac{\partial v}{\partial x})+\bar{C_{36}^{k}}\frac{\partial w}{\partial z}(\frac{\partial u}{\partial y}+\frac{\partial v}{\partial x})]\\ & +2(\bar{C_{12}^{k}}\frac{\partial u}{\partial x}\frac{\partial v}{\partial y}+\bar{C_{13}^{k}}\frac{\partial u}{\partial x}\frac{\partial w}{\partial z}+\bar{C_{23}^{k}}\frac{\partial v}{\partial y}\frac{\partial w}{\partial z})+2\bar{C_{45}^{k}}(\frac{\partial u}{\partial z}+\frac{\partial w}{\partial x})(\frac{\partial w}{\partial y}+\frac{\partial v}{\partial z})\}dxdydz \end{array}$$

(36) $$\begin{array}{ll} U_{sp} & =\frac{1}{2}\int_{0}^{h}\int_{0}^{b}\left\{{\left[k_{x0}^{u}u^{2}+k_{x0}^{v}v^{2}+k_{x0}^{w}w^{2}\right]}_{x=0}+{\left[k_{xa}^{u}u^{2}+k_{xa}^{v}v^{2}+k_{xa}^{w}w^{2}\right]}_{x=a}\right\}dydz\\ & +\frac{1}{2}\int_{0}^{h}\int_{0}^{a}\left\{{\left[k_{y0}^{u}u^{2}+k_{y0}^{v}v^{2}+k_{y0}^{w}w^{2}\right]}_{y=0}+{\left[k_{yb}^{u}u^{2}+k_{yb}^{v}v^{2}+k_{yb}^{w}w^{2}\right]}_{y=b}\right\}dxdz \end{array}$$

(37) $$W_{e}={∭}_{V}(f_{u}u+f_{v}v+f_{w}w)dV$$

where $T_{p}$ is the kinetic energy of the entire plate, $T_{h}$ is the kinetic energy of the cutout part, $U_{p}$ is the strain energy of the entire plate, $U_{h}$ is the strain energy of the cutout part, $U_{sp}$ is the potential energy stored in the boundary springs, and $W_{e}$ is the work done by the external force.

The basic idea to solve the vibration of a plate with cutouts is to subtract the energies of the cutouts part from the total energy of the plate without cutouts. In that sense, the total energy of a plate with a cutout is express as Equation (38), a plate without cutouts can be seen as a special case of a plate with cutouts, the total energy of a plate without cutouts is expressed as Equation (39)

(38) $$L=L_{p}-L_{h}=(T_{p}-T_{h})-(U_{p}-U_{h})-U_{sp}+W_{e}$$

(39) $$L_{p}=T_{p}-U_{p}-U_{sp}+W_{e}$$

### 2.4. Admissible Displacement Functions

The selection of the admissible displacement functions has a significant effect on the convergence and efficiency of the results. According to [56], the author expressed the displacement field functions as Fourier series with auxiliary polynomials, the integration process is complicated due to the non-orthogonality between Fourier series and polynomials. With the increase of the cutout number, the solution process will become more and more complex, therefore, the selection of the admissible displacement functions is significant to reduce the complexity of the calculation. In this paper, the admissible displacement functions are expressed in the form of complete trigonometric Fourier series expansions due to the orthogonality and completeness, as well as the excellent stability in numerical calculations when using the Rayleigh-Ritz method. The admissible functions are expressed as the component segment standard cosine Fourier series functions, and the potential derivative discontinuities of the displacements at the boundary are transferred to the added auxiliary trigonometric terms. The admissible displacement functions are expressed as three variables separated along the *x*, *y*, and *z* directions.

(40) $$\begin{array}{ll} \left\{\begin{array}{c} U(x,y,z)\\ V(x,y,z)\\ W(x,y,z) \end{array}\right\} & =\sum_{m=0}^{M}\sum_{n=0}^{N}\sum_{q=0}^{Q}\left\{\begin{array}{c} A_{mnq}\\ B_{mnq}\\ C_{mnq} \end{array}\right\}\cos\lambda_{m}x\cos\lambda_{n}y\cos\lambda_{q}z\\ & +\sum_{m=0}^{M}\sum_{n=0}^{N}\left[\left\{\begin{array}{c} a_{u\_z}\\ a_{v\_z}\\ a_{w\_z} \end{array}\right\}\xi_{1c}(z)+\left\{\begin{array}{c} b_{u\_z}\\ b_{v\_z}\\ b_{w\_z} \end{array}\right\}\xi_{2c}(z)\right]\cos\lambda_{m}x\cos\lambda_{n}y\\ & +\sum_{m=0}^{M}\sum_{q=0}^{Q}\left[\left\{\begin{array}{c} a_{u\_y}\\ a_{v\_y}\\ a_{w\_y} \end{array}\right\}\xi_{1b}(y)+\left\{\begin{array}{c} b_{u\_y}\\ b_{v\_y}\\ b_{w\_y} \end{array}\right\}\xi_{2b}(y)\right]\cos\lambda_{m}x\cos\lambda_{q}z\\ & +\sum_{n=0}^{N}\sum_{q=0}^{Q}\left[\left\{\begin{array}{c} a_{u\_x}\\ a_{v\_x}\\ a_{w\_x} \end{array}\right\}\xi_{1a}(x)+\left\{\begin{array}{c} b_{u\_x}\\ b_{v\_x}\\ b_{w\_x} \end{array}\right\}\xi_{2a}(x)\right]\cos\lambda_{n}y\cos\lambda_{q}z \end{array}$$

where $\lambda_{m}=m\pi /a$, $\lambda_{n}=n\pi /b$, $\lambda_{q}=q\pi /h$, and $A_{mnq}$, $B_{mnq}$, and $C_{mnq}$ are the unknown Fourier coefficients of the three-dimensional Fourier series expansions for the admissible displacement functions, *M*, *N*, and *Q* are the truncation numbers with respect to variables *x*, *y*, and *z*, respectively. The supplementary functions are defined as

(41) $$\xi_{1a}(x)=\frac{1}{2}\left[\sin(2\pi x/a)+\sin(\pi x/a)\right],\xi_{2a}(x)=\frac{1}{2}\left[\cos(3\pi x/2a)-\cos(\pi x/2a)\right]$$

(42) $$\xi_{1b}(y)=\frac{1}{2}\left[\sin(2\pi y/b)+\sin(\pi y/b)\right],\xi_{2b}(y)=\frac{1}{2}\left[\cos(3\pi y/2b)-\cos(\pi y/2b)\right]$$

(43) $$\xi_{1c}(z)=\frac{1}{2}\left[\sin(2\pi z/h)+\sin(\pi z/h)\right],\xi_{2c}(z)=\frac{1}{2}\left[\cos(3\pi z/2h)-\cos(\pi y/2h)\right]$$

It is easy to verify that

(44) $$\xi_{1a}(0)=\xi_{1a}(a)=\xi_{1a}^{’}(a)=0,\xi_{1a}^{’}(0)=1$$

(45) $$\xi_{2a}(0)=\xi_{2a}(a)=\xi_{2a}^{’}(0)=0,\xi_{2a}^{’}(a)=1$$

$\xi_{1b}$, $\xi_{2b}$, $\xi_{1c}$ and $\xi_{2c}$ have the same expression as $\xi_{1a}$ and $\xi_{2a}$. The six supplementary functions are introduced to solve the potential discontinuities of displacement functions of three-dimensional plate structure at the boundary conditions. The displacement functions can satisfy arbitrary boundary conditions and will significantly improve the convergence of the solution compared with the polynomials and reduce the calculation time due to the orthogonality of the trigonometric Fourier series.

### 2.5. Solution Procedure

In order to obtain the exact solution of the governing equations and boundary conditions of the plate with a cutout, the Hamilton’s principle is applied. Substituting the admissible displacement functions Equation (40) to Equation (43) into the Lagrangian energy functions Equation (32) to Equation (39), and minimizing the function *L* with respect to the unknown Fourier coefficients of the admissible displacement functions, one can easily obtain

(46) $$\frac{\partial L}{\partial X}=0$$

The Equations of motion for the plate with a cutout can be yielded and are summed up in the matrix form as

(47) $$(K-\omega^{2}M)X=0$$

where *K* is the stiffness matrix of the plate and *M* is the mass matrix. The stiffness matrix and the mass matrix both are symmetric and they can be expressed as

(48) $$K=\left[\begin{array}{ccc} K_{uu} & K_{uv} & K_{uw}\\ K_{uv}^{T} & K_{vv} & K_{vw}\\ K_{uw}^{T} & K_{vw}^{T} & K_{ww}^{} \end{array}\right]$$

(49) $$M=\left[\begin{array}{ccc} M_{uu} & 0 & 0\\ 0 & M_{vv} & 0\\ 0 & 0 & M_{uv}^{} \end{array}\right]$$

*X* is the column vector which contains the unknown Fourier coefficients. The natural frequencies and mode shapes of laminated composite rectangular plate with cutouts can be obtained by solving the eigenvalues of Equation (47). Due to the large number of formula terms of *K* and *M*, the detailed calculation expressions are not given here.

## 3. Results and Discussion

In this part, numerical examples pertaining to the free vibration analysis of multilayered laminated composite rectangular or square plates with/without cutouts are presented in order to demonstrate the validity and efficiency of the described method. The results are compared with the solutions available in the existing literature and the finite element analysis. The effect of eccentricity of cutout positions and cutout sizes are studied for free vibration behavior with different boundary conditions. Numerical results are obtained with different layered laminations of symmetric and antisymmetric cross-ply and angle-ply.

Four groups of springs are introduced to simulate different kinds of boundary conditions, including free (F), simply supported (S), clamped supported (C), and elastic supported (E) boundary conditions, the stiffness of the springs are given in Table 1. Unless other stated, all layers of the plate have equal thickness and the same material parameters. The geometrical dimensions and material properties of the plate are given as follows: b=1 m, b/a=1, h/b=0.2; $E_{1}=40E_{2}$, $E_{2}=E_{3}=2$ GPa, $G_{23}/E_{2}=0.5$, $G_{12}=G_{13}=0.6E_{2}$, $\mu_{12}=\mu_{12}=\mu_{12}=0.25$, ρ=1500 kg/m^3^. The non-dimensional frequency parameters are expressed as $\Omega =\omega b^{2}\sqrt{\rho /E_{2}h^{2}}$.

### 3.1. Validity and Convergence of The Method

In this section, some numerical examples are carried out to verify the validity and the convergence of the proposed method in terms of thickness, boundary conditions, and cutout sizes. The accuracy of the method is confirmed by a comparison study with the data published in the literature of rectangular plate with/without considering cutouts, the data are given in Table 2 and Table 3. The symbol ‘-’ in Table 2 and Table 3 indicate that the frequencies were not considered in the reference work.

First, for the case of plate without cutouts, the first six natural frequencies of a three-layered, cross-ply (0°/90°/0°) laminated square plate without cutouts under simply supported boundary conditions are presented in Table 2. Three kinds of thickness-length ratios, i.e., h/a *=* 0.01, 0.1, 1 corresponding to thin, moderately thick, and thick plates are performed, whereas h/a=1 represents a solid cube, respectively. From Table 2, it is obvious that the proposed method has fast convergence behavior. The maximum error of the 9 × 9 × 9 truncated configuration compared with [44] is 0.086%, which proves the validity and accuracy of this method. In the following examples, the Fourier series is truncated to M × N × Q = 9 × 9 × 9. In Table 3, the first six natural frequencies of a four-layered, angle-ply (45°/−45°/45°/−45°) laminated square plate without cutouts are presented, and the FFFF, SFSF, FFCF boundary conditions with different thickness-length ratios are performed in the comparison. The geometrical dimensions and material properties are the same as for Table 2. It can be seen from the table that the maximum error with the first-four natural frequencies provided by Qu et al. [44] is 0.155%, and the present results are in good agreement with those results of Ref. [44].

Second, in order to verify the reliability of solving the vibration characteristics of a plate with cutouts, a two-layered, cross-ply (0°/90°) laminated square plate with central square cutouts or rectangular cutouts under simply supported boundary condition has been considered, and the first six natural frequency parameters are performed in Table 4. The results of the proposed method are compared with those obtained by Sheikh et al. [57]. The geometrical dimensions and material properties of the plate are given as follows: b=1 m, b/a=1, h/b=0.01; $E_{1}=25E_{2}$, $E_{2}=E_{3}=2$ GPa, $G_{23}/E_{2}=0.2$, $G_{12}=G_{13}=0.5E_{2}$, $\mu_{12}=\mu_{12}=\mu_{12}=0.25$, ρ=1500 kg/m^3^. From the table, it can be seen that the difference between the data of the proposed method and Sheikh et al. [57] is less than 0.556%, which shows that the present method is able to accurately predict the frequency parameters of a rectangular plate with cutouts. The small differences are related to different laminated plate theory and solution program methods.

### 3.2. Effect of Eccentricity of Cutout Position

In this section, the effect of eccentricity of cutout positions is studied for antisymmetric cross-ply (0°/90°/0°/90°) laminated rectangular plate with CCCC (four edges clamped) boundary condition. The geometry dimensions are: h/b=0.2, b=1 m, a/b=2, c/a=0.2, d/b=0.2. The coordinates of the cutout center ratio, thus eccentricity ratio, *x_h_/a* and *y_h_*/*b* are varied from 0.2 to 0.8 along the *x* direction and *y* direction, separately. Table 5 and Table 6 show some of the first 20 non-dimensional frequency parameters compared with the solution of finite element method. The finite element method results are obtained from 3D model by using the software ANSYS Workbench, and the element size is selected as 5 × 5 mm. The present solution agrees well with the results of finite element method, and the data obtained by finite element method are slightly larger than the data obtained by the present method because of the finite element method needs to refine mesh to obtain enough accuracy. The data are symmetric about the geometric centers due to the symmetry of the geometry.

**Figure 2:**
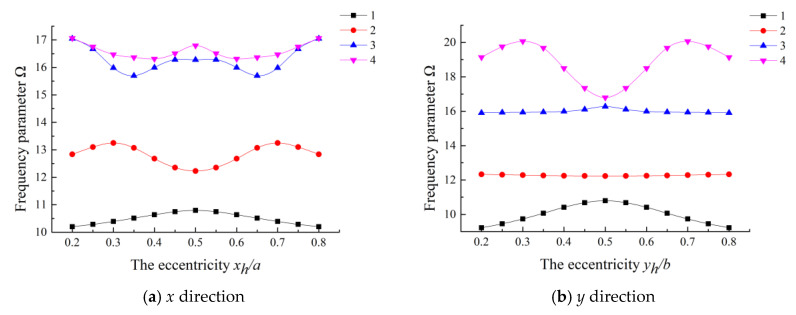
Variation of the frequency parameters for a (0°/90°/0°/90°) laminated rectangular plate with different cutout eccentricity ratios.

In Figure 2, variation of the lowest four order frequency parameters against the cutout position ratio are shown for the eccentricity in both *x* and *y* directions. From Figure 2a, we can see that the frequency firstly increases and then decreases for the first and second order frequency parameters when the eccentricity in the *x* direction goes from 0.2 to 0.5, while the third and fourth orders have reverse behavior, respectively. From Figure 2b, there is little variation in the second and third orders with the eccentricity ratio varied in the *y* direction. However, for the fourth order, the frequency parameter has gone through the process of decrease after going up for *y_h_/b* in the range of 0.2–0.5. For the first order, the fundamental frequency increases and then declines both in the *x* direction and *y* direction, and reaches its wave crest at 0.5. It can conclude that, for a rectangular plate with different aspect ratios, the cutout positions have different effects on the vibration characteristics. Nevertheless, for a square plate with a square cutout, it is conceivable that the frequency parameter variation is consistent in the *x* direction and *y* direction.

### 3.3. Effect of Cutout Size

**Figure 3:**
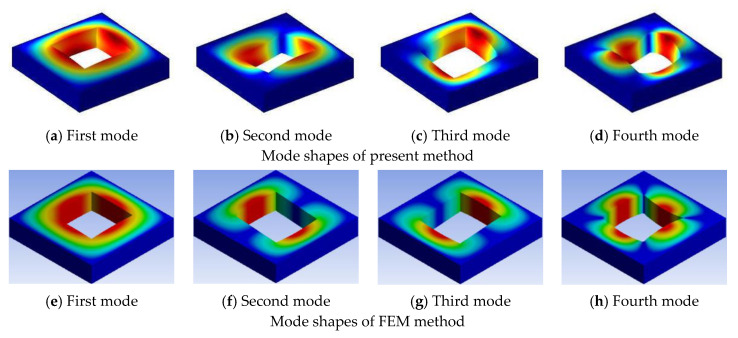
First four mode shapes of (0°/90°/0°/90°) laminated square plate with CCCC boundary condition for a cutout size ratio of 0.5.

**Figure 4:**
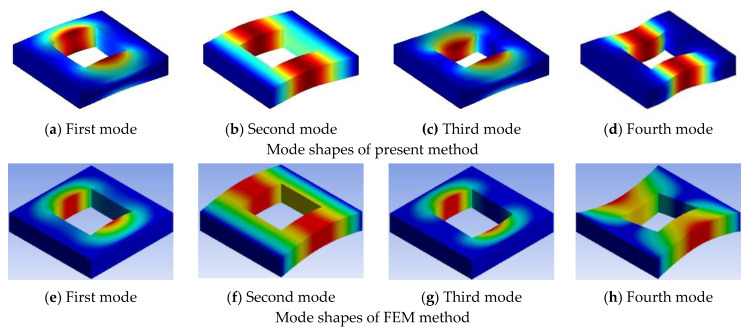
First four mode shapes of (0°/90°/90°/0°) laminated square plate with CSCS boundary condition for a cutout size ratio of 0.5.

**Figure 5:**
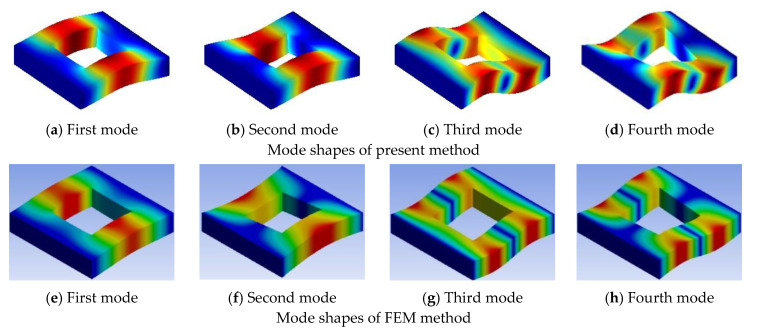
First four mode shapes of (45°/−45°/45°/−45°) laminated square plate with CFCF boundary condition for a cutout size ratio of 0.5.

Three examples are selected to demonstrate the effect of the cutout size with different boundary conditions, the cutout size ratio is varied from 0 to 0.8. In Table 7, some of the first 20 non-dimensional frequency parameters for the symmetric cross-ply (0°/90°/0°/90°) lamination with different cutout sizes under CCCC boundary conditions are examined. From Table 7, it can be seen that increasing of the cutout size of the plate increases the fundamental frequency parameters. The increasing trend is due to the introduction of the cutout; however, the loss of mass gets more significance over the loss of stiffness and hence the frequency parameters increase. Table 8 shows the data of antisymmetric cross-ply (0°/90°/90°/0°) laminated square plate with different cutout sizes under CSCS boundary condition, and Table 9 for angle-ply (45°/−45°/45°/−45°) laminated square plate with different cutout sizes under CFCF boundary condition. It is evident that from Table 8 and Table 9, the fundamental frequency parameters increase and then decrease when the cutout size ratio is varied from 0 to 0.8. The typical mode shapes corresponding to the first four orders of vibration for the plate compared with those obtained from the finite element method are given in Figure 3, Figure 4 and Figure 5.

### 3.4. Effect of Lamination

**Figure 6:**
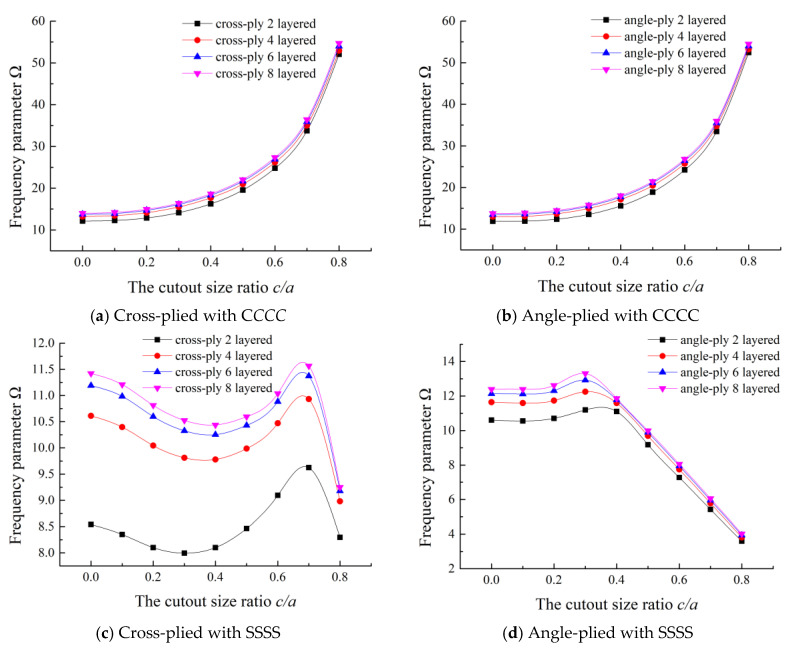
Fundamental frequency parameters of different laminations with various cutout sizes.

To further study the effect of the number of layers on the frequency characteristic, the fundamental frequency parameters of two-layered, three-layered, four-layered, six-layered, and eight-layered, cross-ply and angle-ply plates with different cutout sizes are presented, the data are given in Table A1. Figure 6 shows the fundamental frequency parameters of cross-ply and angle-ply laminations with even numbers of layers under CCCC and SSSS boundary conditions. From Figure 6, it can be noticed that the frequency parameters increase with the increase of the number of the layers for both cross-ply and angle-ply. From Figure 6a,b, it is obvious that for CCCC boundary condition, the frequency parameters increase with the increase of cutout size ratio, and when the cutout size ratio exceeds 0.6, the frequency parameters increase sharply. However, it is somewhat complicated for SSSS boundary condition. From Figure 6c, for cross-ply lamination, the trend of frequency parameters declines first and then rises, when the cutout size ratio reaches a certain value which is respectively equal to 0.7, the frequency parameter decreases sharply. For angle-ply lamination, there is only a little bit of an upward trend when the cutout size ratio is less than 0.3, while after that, the frequency parameter trend declines with the increase of cutout sizes.

### 3.5. Effect of Boundary Condition

Next, to research the effect of boundary conditions, a four-layered, symmetric, and antisymmetric composite plate under six groups of boundary conditions, i.e., CCCC, CSCC, CSSC, CSCS, CSSS, and SSSS are considered. The fundamental frequency parameters are calculated for various cutout sizes and the data are detailed in Table 10.

It can be seen that the fundamental frequency parameters of antisymmetric structures are slightly larger than those of symmetric structures and the maximum frequency parameters are under the CCCC boundary conditions. Comparing the boundary conditions of CCCC and CSCC, for the same cutout size ratios, the boundary constrain is released from clamp supported to simply supported, the stiffness becomes smaller and the mass remains unchanged, thus the frequency parameter decreases both for symmetric and antisymmetric structures, cross-ply and angle-ply laminations. For different boundary conditions, with the increase of the cutout sizes, both of the mass matrix and the stiffness matrix get smaller, the frequency parameters tend to rise or drop. When the frequency parameters increase, mainly because the loss mass is more significant than the loss of stiffness, whereas the reason is reverse for the frequency parameters decreasing.

### 3.6. Effect of Layer Fiber Direction Angle

**Figure 7:**
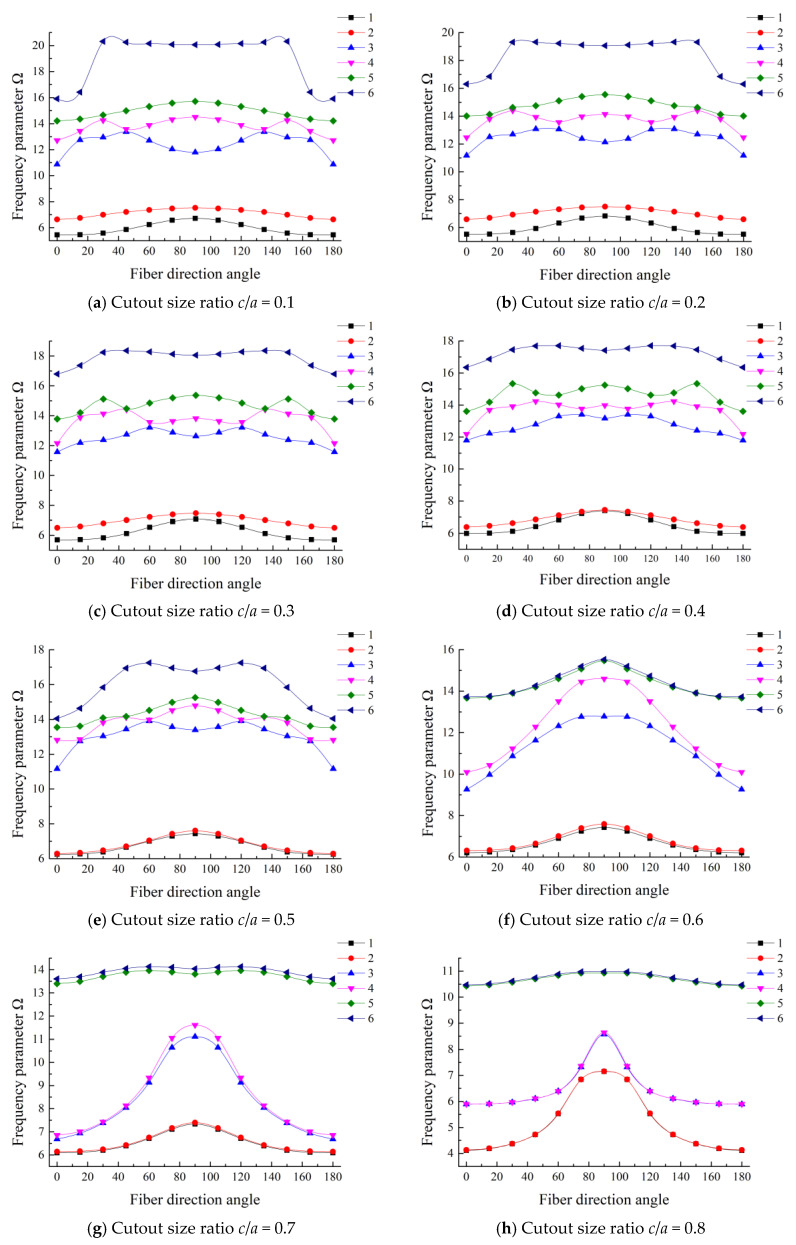
The first six frequency parameters of different fiber orientations with various cutout sizes.

The effect of layer fiber direction angle on the frequency parameters of laminated composite plate with cutouts is studied in the following section. The detailed data are given in Table A2. The first six frequency parameters of a three-layered angle-ply (0°/ϑ°/0°) square plate with a center cutout under CFCF boundary condition are investigated, where ϑ is the fiber angle in the range of 0°–90°. In Figure 7, the curves of frequency parameters changing with the fiber orientations under various cutout sizes are plotted. The fiber direction angle is varied from 0° to 180° with a step of 15°. All of the curves are symmetric about ϑ = 90°, and the trend of frequency parameters is not changing in the same way with the increase of cutout size ratio. The fundamental frequency parameters increase and then decrease with the increase of the cutout size ratio. The trend can be regrouped in three groups according to the performance: Group I, a smaller cutout size ratio, where 0 ≤ *c*/*a* ≤ 0.2; Group II, 0.2 < *c*/*a* ≤ 0.5; Group III, a larger cutout size ratio, where 0.5 < *c*/*a* ≤ 0.8. The trend in each group is generally consistent. It can be observed that the increment of the fiber direction angle from 0° to 90° results in the increase of the lowest two frequency parameters of the plate. For Group III, the frequency parameters climb up with the fiber direction angle changed from 0° to 90°. When the cutout size ratio is small, the trend of the lower-order modal is simpler, but for higher-order modal, the trend is more complex. This is mainly because the bending stiffness plays a dominant role for the lower-order modal, while for the higher-order modal, the bending and shear deformation are coupled with each other, which make the vibration behavior more complex.

## 4. Conclusions

In this paper, a unified three-dimensional solution is provided to study the free vibration characteristics of laminated composite rectangular plate with/without cutouts by using the energy principles in conjunction with Rayleigh-Ritz method. The energy equations of the plate and the cutouts which are based on the three-dimensional elasticity theory are established independently. The governing equation was obtained by subtracting the energies of cutouts from the total energies of the entire plate. The displacement field is represented as a standard three-dimensional trigonometric cosine Fourier series and modified auxiliary Fourier series which can satisfy the boundary conditions and reduce the computational complexity. The three-dimensional solution is obtained by using the Hamilton’s principle to solve the unknown Fourier coefficients. The advantage of this method is that the unified model can simulate the different boundary conditions by changing the values of spring stiffness and there is no need to establish various models separately. For the free vibration characteristics of laminated composite rectangular plate with/without cutouts under various boundary conditions, the accuracy of this method is better than the results of the literature and finite element analysis. Furthermore, some numerical examples are carried out to show the performance of the proposed method and we can arrive at the following conclusions:

First, the frequency parameters of laminated composite square plate with/without cutouts under various boundary conditions are calculated and compared with those available in the literature. It can be seen from the comparison that the proposed method is accurate in predicting the vibration characteristics of plates with cutouts.

Second, the influence of some key parameters, such as the cutout positions, the cutout sizes, the boundary conditions, the laminations, and the fiber orientations on the frequency parameters are discussed. The cutout positions have a significant effect on the frequency parameters, and the maximum fundamental frequency occurs when the cutout is located in the center of plate. From the variations of the cutout size ratio, it can be concluded that with the increase of cutout size ratio under various boundary conditions, the loss of mass and stiffness play a different role in determining the frequency parameters. When the total thickness is constant, the frequency parameter grows as the number of the layers increases. The fundamental frequency parameters climb up as the fiber direction angle varies from 0°–90°.

## Figures and Tables

**Table 1:** Values of spring stiffness taken by different boundary conditions

BC	*k_u_*	*k_v_*	*k_w_*
F	0	0	0
S	0	10^12^	10^12^
C	10^12^	10^12^	10^12^
E	Arbitrary	Arbitrary	Arbitrary

**Table 2:** The first six natural frequencies (Hz) of simply supported cross-ply (0°/90°/0°) laminated square plate with different thickness-length ratios.

*h/a*	M × N × Q	First	Second	Third	Fourth	Fifth	Sixth
0.01	4 × 4 × 4	34.838	51.127	92.728	131.155	141.307	159.719
	6 × 6 × 6	34.631	49.611	85.157	129.977	137.533	141.870
	8 × 8 × 8	34.602	49.376	84.654	129.823	137.053	139.008
	9 × 9 × 9	34.602	49.375	84.653	129.823	137.052	139.004
	Ref. [44]	34.600	49.360	84.584	129.815	-	-
	Error (%)	0.006	0.030	0.082	0.006	-	-
0.05	4 × 4 × 4	160.865	233.566	398.244	447.248	447.261	507.561
	6 × 6 × 6	160.659	231.778	389.900	447.218	447.220	506.404
	8 × 8 × 8	160.633	231.537	388.783	447.214	447.214	506.265
	9 × 9 × 9	160.631	231.511	388.661	447.214	447.214	506.251
	Ref. [44]	160.627	-	-	447.203	-	-
	Error (%)	0.002	-	-	0.002	-	-
0.1	4 × 4 × 4	270.174	399.265	447.231	447.239	647.363	687.064
	6 × 6 × 6	270.100	398.553	447.215	447.216	643.710	686.606
	8 × 8 × 8	270.093	398.480	447.214	447.214	643.353	686.559
	9 × 9 × 9	270.083	398.367	447.214	447.214	642.900	686.492
	Ref. [44]	270.076	398.330	447.192	447.203	-	-
	Error (%)	0.003	0.009	0.005	0.002	-	
1	4 × 4× 4	447.230	447.239	526.146	614.985	620.787	721.754
	6 × 6× 6	447.215	447.216	526.105	614.963	620.782	721.722
	8 × 8× 8	447.214	447.214	526.101	614.961	620.781	721.719
	9 × 9 × 9	447.214	447.214	525.195	614.949	620.769	720.335
	Ref. [44]	447.192	447.203	524.744	614.932	-	-
	Error (%)	0.005	0.002	0.086	0.003	-	-

**Table 3:** First six natural frequencies (Hz) of angle-ply (45°/−45°/45°/−45°) laminated square plate with different thickness-length ratios and different boundary conditions.

*h/a*	BC	Modes	First	Second	Third	Fourth	Fifth	Sixth
0.05	FFFF	Present	73.571	152.467	197.424	286.400	268.400	406.345
		Ref. [44]	73.607	152.547	197.472	286.508	-	-
		Error (%)	0.049	0.052	0.024	0.038	-	-
0.2	SFSF	Present	178.958	351.236	547.335	597.878	629.600	632.456
		Ref. [44]	178.974	351.194	547.802	598.081	-	-
		Error (%)	0.009	0.012	0.085	0.034	-	-
0.5	FFCF	Present	132.031	249.995	282.025	396.238	484.985	593.017
		Ref. [44]	131.977	249.609	282.040	396.234	-	-
		Error (%)	0.041	0.155	0.005	0.001	-	-

BC: Boundary Conditions.

**Table 4:** First six frequency parameters Ω of cross-ply (0°/90°) laminated square plate with different cutout sizes under simply supported boundary condition

Cutout Size	Modes	1	2	3	4	5	6
0.2a × 0.2a	Present	9.118	25.482	25.482	38.042	54.014	60.815
	Ref. [57]	9.116	25.500	25.515	38.045	54.033	60.646
	Error (%)	0.022	0.071	0.129	0.008	0.035	0.279
0.4a × 0.4a	Present	9.1004	20.398	20.399	35.495	44.505	61.701
	Ref. [57]	9.093	20.409	20.429	35.483	44.599	61.713
	Error (%)	0.081	0.054	0.147	0.034	0.211	0.019
0.6a × 0.6a	Present	11.135	18.572	18.572	32.934	34.373	53.263
	Ref. [57]	11.113	18.536	18.549	32.941	34.271	53.137
	Error (%)	0.198	0.194	0.124	0.021	0.298	0.237
0.4a × 0.2a	Present	8.809	21.181	24.750	37.029	51.958	62.081
	Ref. [57]	8.805	21.234	24.759	37.039	51.987	62.058
	Error (%)	0.045	0.250	0.036	0.027	0.056	0.037
0.6a × 0.2a	Present	8.534	15.830	25.379	34.603	51.547	60.234
	Ref. [57]	8.527	15.842	25.362	34.628	51.527	60.137
	Error (%)	0.082	0.076	0.067	0.072	0.039	0.161
0.8a × 0.4a	Present	9.662	11.762	27.305	31.078	50.807	60.364
	Ref. [57]	9.648	11.788	27.216	31.060	50.526	60.247
	Error (%)	0.145	0.221	0.327	0.058	0.556	0.194

**Table 5:** Some of the first 20 frequency parameters Ω of cross-ply (0°/90°/0°/90°) laminated rectangular plate with different cutout positions in the x direction under CCCC boundary condition.

*x_h_/a*	Modes	1	2	3	4	5	6	10	20
0.2	Present	10.199	12.835	17.048	17.063	19.771	20.728	29.518	38.134
	FEM	10.203	12.844	17.077	17.084	19.787	20.757	29.565	38.226
	Error(%)	0.058	0.082	0.166	0.124	0.077	0.133	0.160	0.243
0.25	Present	10.286	13.103	16.670	16.753	19.852	20.337	28.653	38.456
	FEM	10.293	13.112	16.703	16.768	19.867	20.357	28.713	38.541
	Error(%)	0.060	0.074	0.210	0.091	0.077	0.101	0.209	0.219
0.3	Present	10.393	13.249	15.988	16.469	19.966	20.494	29.659	38.820
	FEM	10.398	13.255	16.004	16.502	19.982	20.514	29.702	38.896
	Error(%)	0.058	0.062	0.106	0.200	0.078	0.094	0.144	0.197
0.35	Present	10.512	13.072	15.697	16.364	20.102	20.811	29.822	38.666
	FEM	10.518	13.081	15.713	16.395	20.118	20.831	29.860	38.749
	Error(%)	0.058	0.066	0.102	0.181	0.080	0.098	0.129	0.215
0.4	Present	10.637	12.681	15.995	16.310	20.245	20.786	28.496	38.112
	FEM	10.644	12.690	16.008	16.341	20.263	20.811	28.559	38.181
	Error(%)	0.057	0.079	0.091	0.187	0.085	0.111	0.219	0.181
0.45	Present	10.747	12.352	16.283	16.509	20.339	20.366	29.082	38.858
	FEM	10.753	12.363	16.312	16.522	20.359	20.384	29.131	38.908
	Error(%)	0.055	0.079	0.171	0.077	0.105	0.082	0.168	0.125
0.5	Present	10.794	12.229	16.276	16.795	20.017	20.418	29.740	39.062
	FEM	10.800	12.238	16.303	16.806	20.037	20.433	29.773	39.138
	Error(%)	0.054	0.080	0.168	0.067	0.107	0.081	0.115	0.190

**Table 6:** Some of the first 20 frequency parameters Ω of cross-ply (0°/90°/0°/90°) laminated rectangular plate with different cutout positions in the y direction under CCCC boundary condition.

*y_h_/b*	Modes	1	2	3	4	5	6	10	20
0.2	Present	9.223	12.331	15.909	19.135	20.207	20.679	29.183	38.727
	FEM	9.234	12.339	15.920	19.154	20.226	20.692	29.234	38.795
	Error(%)	0.118	0.059	0.068	0.099	0.093	0.064	0.172	0.176
0.25	Present	9.449	12.307	15.934	19.754	20.179	20.668	28.875	38.795
	FEM	9.461	12.315	15.945	19.774	20.201	20.683	28.926	38.874
	Error(%)	0.127	0.069	0.073	0.104	0.108	0.071	0.176	0.201
0.3	Present	9.736	12.283	15.941	20.063	20.138	20.636	27.677	38.588
	FEM	9.748	12.292	15.953	20.084	20.161	20.652	27.729	38.674
	Error(%)	0.123	0.078	0.079	0.106	0.114	0.076	0.188	0.220
0.35	Present	10.069	12.262	15.951	19.679	20.094	20.576	26.227	38.615
	FEM	10.079	12.271	15.967	19.701	20.115	20.592	26.259	38.699
	Error(%)	0.100	0.077	0.097	0.110	0.107	0.082	0.120	0.216
0.4	Present	10.408	12.244	15.992	18.502	20.053	20.502	27.751	38.772
	FEM	10.416	12.255	16.010	18.527	20.076	20.519	27.792	38.853
	Error(%)	0.085	0.083	0.112	0.136	0.113	0.083	0.147	0.207
0.45	Present	10.683	12.233	16.111	17.348	20.026	20.440	29.159	38.945
	FEM	10.690	12.242	16.133	17.368	20.048	20.458	29.199	39.024
	Error(%)	0.068	0.080	0.139	0.113	0.107	0.085	0.135	0.201
0.5	Present	10.794	12.229	16.276	16.795	20.017	20.417	29.739	39.063
	FEM	10.800	12.238	16.304	16.806	20.038	20.434	29.773	39.137
	Error(%)	0.054	0.080	0.168	0.067	0.107	0.081	0.115	0.190

**Table 7:** Some of the first 20 frequency parameters Ω of cross-ply (0°/90°/0°/90°) laminated square plate with different cutout sizes under CCCC boundary condition.

Cutout Size Ratio *c*/*a*	1	2	3	4	5	6	10	20
0	13.234	21.120	21.120	26.947	30.878	30.913	41.044	57.042
0.10	13.419	20.605	20.605	26.881	30.614	31.571	39.720	57.200
0.20	14.140	19.494	19.495	26.587	29.497	33.671	39.164	56.613
0.30	15.514	19.091	19.091	26.086	27.051	33.985	41.105	57.545
0.40	17.688	19.988	19.988	25.324	26.051	32.834	44.407	57.943
0.50	20.997	22.438	22.438	25.701	27.342	32.351	45.462	59.197
0.60	26.177	27.045	27.046	28.862	30.875	34.195	45.346	65.597
0.70	35.012	35.492	35.492	36.369	38.423	40.358	48.670	73.990
0.80	52.924	53.139	53.139	53.460	55.321	56.214	61.651	82.004

**Table 8:** Some of the first 20 frequency parameters Ω of cross-ply (0°/90°/90°/0°) laminated square plate with different cutout sizes under CSCS boundary condition.

Cutout Size Ratio *c*/*a*	1	2	3	4	5	6	10	20
0	11.905	12.167	18.111	22.018	24.335	26.026	36.126	49.930
0.10	11.976	12.287	17.780	21.291	23.883	25.970	35.029	49.397
0.20	12.373	12.634	16.805	19.735	22.973	25.687	34.145	47.632
0.30	13.102	13.172	15.724	19.137	22.353	25.098	34.531	48.733
0.40	13.808	13.883	14.946	20.021	22.469	23.377	32.530	48.618
0.50	14.181	14.311	14.404	19.452	22.392	23.482	31.474	47.928
0.60	13.875	13.892	14.290	16.552	25.164	25.580	32.823	49.947
0.70	13.321	13.326	13.470	14.197	25.914	26.365	36.759	50.614
0.80	11.814	11.937	12.649	12.654	23.758	24.137	37.241	58.710

**Table 9:** Some of the first 20 frequency parameters Ω of angle-ply (45°/−45°/45°/−45°) laminated square plate with different cutout sizes under CFCF boundary condition.

Cutout Size Ratio *c*/*a*	1	2	3	4	5	6	10	20
0	8.574	11.202	17.488	17.804	20.006	26.106	33.149	43.589
0.10	8.573	11.090	17.304	17.753	19.851	25.521	33.105	42.808
0.20	8.669	10.722	16.726	17.917	19.506	23.763	32.570	40.828
0.30	8.888	10.187	16.267	18.420	18.938	22.160	31.736	41.297
0.40	9.083	9.655	16.427	18.219	19.333	21.305	28.056	44.490
0.50	9.041	9.190	16.959	17.611	20.287	20.619	24.274	44.692
0.60	8.702	8.719	16.479	16.491	17.347	17.426	24.322	42.414
0.70	8.158	8.159	10.687	10.702	16.639	16.649	21.433	38.076
0.80	5.900	5.902	7.498	7.499	12.260	12.262	16.283	36.992

**Table 10:** Fundamental frequency parameters Ω of square plate for various cutout size ratio with different boundary conditions.

Laminations	BC	Cutout Size Ratio								
		0	0.1	0.2	0.3	0.4	0.5	0.6	0.7	0.8
(0°/90°)_2_	CCCC	13.234	13.419	14.140	15.514	17.688	20.997	26.177	35.012	52.924
	CSCC	12.522	12.595	12.979	13.603	14.030	13.970	13.614	13.177	12.059
	CSSC	11.783	11.757	11.820	11.930	11.905	11.833	11.892	11.935	10.401
	CSCS	11.972	11.965	12.168	12.700	13.392	13.779	13.557	13.092	11.992
	CSSS	11.209	11.087	10.954	10.918	10.918	10.982	11.227	11.463	9.692
	SSSS	10.612	10.399	10.046	9.811	9.778	9.987	10.471	10.934	8.979
0°/90°/90°/0°	CCCC	13.198	13.383	14.105	15.464	17.563	20.618	25.186	33.058	49.937
	CSCC	12.493	12.608	13.094	13.842	14.341	14.292	13.884	13.324	11.887
	CSSC	11.789	11.772	11.848	11.963	11.948	11.912	12.045	12.387	10.260
	CSCS	11.905	11.976	12.373	13.102	13.809	14.181	13.913	13.321	11.814
	CSSS	11.175	11.100	11.070	11.098	11.087	11.132	11.418	12.010	9.577
	SSSS	10.683	10.498	10.177	9.960	9.942	10.175	10.716	11.576	8.896
(45°/−45°)_2_	CCCC	13.008	13.100	13.680	14.969	17.120	20.458	25.727	34.761	53.203
	CSCC	12.584	12.634	13.069	14.015	15.305	16.594	17.696	18.202	12.297
	CSSC	12.234	12.248	12.560	13.277	14.293	15.555	16.231	11.602	7.088
	CSCS	12.187	12.205	12.546	13.386	14.716	16.321	17.657	18.203	12.296
	CSSS	11.903	11.887	12.112	12.734	13.738	15.107	12.750	8.944	5.514
	SSSS	11.641	11.595	11.736	12.250	11.596	9.695	7.746	5.782	3.814
45°/−45°/−45°/45°	CCCC	12.898	12.992	13.576	14.870	17.028	20.379	25.670	34.731	53.196
	CSCC	12.470	12.522	12.953	13.883	15.128	16.372	17.483	18.026	11.939
	CSSC	12.175	12.062	12.340	12.963	13.784	14.837	16.183	11.358	6.928
	CSCS	12.072	12.090	12.423	13.244	14.545	16.114	17.446	18.026	11.939
	CSSS	11.780	11.762	11.968	12.545	13.469	14.701	12.524	8.750	5.394
	SSSS	11.502	11.453	11.565	12.030	11.472	9.557	7.611	5.666	3.732

## Appendix A

**Table A1:** Fundamental frequency parameters Ω of square plate with different cutout sizes under different laminations.

BC	Laminations	Cutout Size Ratio								
		0	0.1	0.2	0.3	0.4	0.5	0.6	0.7	0.8
CCCC	0°/90°	12.150	12.275	12.876	14.142	16.251	19.543	24.769	33.761	52.051
	0°/90°/0°	12.730	12.907	13.606	14.918	16.886	19.624	23.752	31.375	48.357
	(0°/90°)_2_	13.234	13.419	14.140	15.514	17.688	20.997	26.177	35.012	52.924
	0°/90°/90°/0°	13.198	13.383	14.105	15.464	17.563	20.618	25.186	33.058	49.937
	(0°/90°)_3_	13.743	13.941	14.683	16.091	18.310	21.678	26.941	35.889	53.979
	(0°/90°)_4_	14.003	14.204	14.954	16.380	18.626	22.033	27.356	36.402	54.685
	45°/−45°	11.888	11.928	12.390	13.543	15.590	18.888	24.216	33.469	52.450
	45°/−45°/45°	12.359	12.454	13.030	14.304	16.439	19.770	25.059	34.169	52.794
	(45°/−45°)_2_	13.008	13.100	13.680	14.969	17.120	20.458	25.727	34.761	53.203
	45°/−45°/−45°/45°	12.898	12.992	13.576	14.870	17.028	20.379	25.670	34.731	53.196
	(45°/−45°)_3_	13.512	13.621	14.214	15.532	17.723	21.115	26.444	35.521	53.974
	(45°/−45°)_4_	13.765	13.875	14.473	15.805	18.021	21.447	26.823	35.964	54.498
SSSS	0°/90°	8.539	8.351	8.100	7.994	8.101	8.463	9.093	9.624	8.297
	0°/90°/0°	10.234	10.082	9.806	9.630	9.653	9.935	10.534	11.404	8.723
	(0°/90°)_2_	10.612	10.399	10.046	9.811	9.778	9.987	10.471	10.934	8.979
	0°/90°/90°/0°	10.683	10.498	10.177	9.960	9.942	10.175	10.716	11.576	8.896
	(0°/90°)_3_	11.192	10.984	10.600	10.327	10.255	10.429	10.883	11.374	9.178
	(0°/90°)_4_	11.422	11.210	10.814	10.525	10.438	10.596	11.038	11.566	9.249
	45°/−45°	10.607	10.560	10.704	11.196	11.108	9.178	7.278	5.420	3.592
	45°/−45°/45°	10.986	10.935	11.009	11.382	11.431	9.515	7.577	5.645	3.725
	(45°/−45°)_2_	11.641	11.595	11.736	12.250	11.596	9.695	7.746	5.782	3.814
	45°/−45°/−45°/45°	11.502	11.453	11.565	12.030	11.472	9.557	7.611	5.666	3.732
	(45°/−45°)_3_	12.134	12.119	12.309	12.926	11.774	9.898	7.951	5.958	3.942
	(45°/−45°)_4_	12.393	12.390	12.617	13.305	11.858	9.994	8.052	6.052	4.016

**Table A2:** First six frequency parameters Ω of angle-ply (0°/ϑ°/0°) square plate with various fiber direction angle varied from 0° to 180° under CFCF boundary condition.

Fiber Angle	Modes	Cutout Size Ratio								
		0	0.1	0.2	0.3	0.4	0.5	0.6	0.7	0.8
0°	1	5.444	5.448	5.516	5.700	5.987	6.254	6.205	6.093	4.115
	2	6.639	6.631	6.588	6.500	6.385	6.284	6.313	6.145	4.134
	3	10.757	10.866	11.170	11.575	11.792	11.162	9.260	6.685	5.892
	4	12.773	12.716	12.467	12.156	12.183	12.817	10.098	6.855	5.897
	5	14.310	14.214	14.010	13.787	13.603	13.542	13.667	13.396	10.426
	6	15.756	15.907	16.302	16.787	16.345	14.047	13.726	13.606	10.473
15°	1	5.459	5.464	5.532	5.715	6.003	6.272	6.241	6.115	4.186
	2	6.758	6.747	6.697	6.593	6.459	6.338	6.332	6.162	4.195
	3	12.809	12.752	12.504	12.196	12.223	12.747	9.975	6.937	5.905
	4	13.304	13.437	13.794	13.885	13.689	12.852	10.434	7.015	5.909
	5	14.466	14.354	14.127	14.209	14.176	13.616	13.727	13.496	10.472
	6	16.252	16.415	16.845	17.361	16.861	14.634	13.757	13.694	10.513
30°	1	5.576	5.579	5.646	5.831	6.119	6.383	6.355	6.202	4.367
	2	7.006	6.991	6.925	6.794	6.630	6.479	6.428	6.240	4.372
	3	13.014	12.954	12.697	12.382	12.409	13.038	10.869	7.379	5.965
	4	14.116	14.252	14.406	14.138	13.913	13.815	11.234	7.434	5.967
	5	14.785	14.658	14.625	15.118	15.332	14.087	13.902	13.705	10.576
	6	20.766	20.317	19.311	18.237	17.450	15.837	13.918	13.887	10.614
45°	1	5.858	5.859	5.929	6.122	6.410	6.649	6.576	6.393	4.722
	2	7.224	7.208	7.141	7.011	6.851	6.706	6.653	6.428	4.729
	3	13.444	13.371	13.083	12.749	12.793	13.440	11.628	8.043	6.109
	4	13.445	13.575	13.930	14.415	14.231	14.119	12.280	8.136	6.111
	5	15.106	14.991	14.752	14.476	14.759	14.164	14.197	13.891	10.704
	6	20.674	20.252	19.324	18.354	17.682	16.945	14.261	14.050	10.744
60°	1	6.238	6.240	6.324	6.536	6.829	7.008	6.901	6.713	5.526
	2	7.374	7.363	7.314	7.220	7.108	7.042	7.007	6.752	5.541
	3	12.586	12.712	13.064	13.215	13.297	13.903	12.317	9.137	6.391
	4	13.980	13.888	13.560	13.567	14.020	13.985	13.510	9.340	6.393
	5	15.408	15.313	15.103	14.849	14.617	14.522	14.600	13.966	10.835
	6	20.574	20.151	19.221	18.279	17.700	17.250	14.730	14.132	10.879
75°	1	6.569	6.578	6.679	6.913	7.218	7.295	7.244	7.110	6.841
	2	7.477	7.471	7.443	7.391	7.335	7.427	7.391	7.162	6.845
	3	11.921	12.040	12.380	12.884	13.405	13.566	12.768	10.645	7.320
	4	14.428	14.324	13.971	13.632	13.766	14.517	14.446	11.051	7.359
	5	15.661	15.582	15.407	15.197	15.017	14.976	15.076	13.904	10.926
	6	20.511	20.078	19.107	18.125	17.531	16.961	15.196	14.104	10.976
90°	1	6.709	6.722	6.834	7.081	7.397	7.436	7.422	7.335	7.148
	2	7.529	7.525	7.506	7.473	7.444	7.613	7.592	7.397	7.155
	3	11.681	11.797	12.130	12.631	13.165	13.390	12.777	11.108	8.569
	4	14.615	14.505	14.145	13.816	13.983	14.793	14.595	11.606	8.637
	5	15.781	15.710	15.552	15.372	15.238	15.256	15.468	13.813	10.926
	6	20.495	20.059	19.065	18.050	17.412	16.764	15.527	14.041	10.980

## References

[1] Liew K.M., Pan Z.Z., Zhang L.W. (2019). An overview of layerwise theories for composite laminates and structures: Development, numerical implementation and application. Compos. Struct..

[2] Kulkarni P.A., Dhoble A.S., Padole P.M. (2018). A review of research and recent trends in analysis of composite plates. Sadhana-Acad. Proc. Eng. Sci..

[3] Love A.E.I. (1887). The small free vibrations and deformation of a thin elastic shell. Proc. R. Soc. Lond..

[4] Gorman D.J. (1993). Accurate Free Vibration Analysis of the Completely Free Orthotropic Rectangular Plate by the Method of Superposition. J. Sound Vib..

[5] Gorman D.J., Ding W. (2003). Accurate free vibration analysis of completely free symmetric cross-ply rectangular laminated plates. Compos. Struct..

[6] Nallim L.G., Martinez S.H., Grossi R.O. (2005). Statical and dynamical behaviour of thin fibre reinforced composite laminates with different shapes. Comput. Methods Appl. Mech. Eng..

[7] Nallim L.G., Oller S. (2008). An analytical-numerical approach to simulate the dynamic behaviour of arbitrarily laminated composite plates. Compos. Struct..

[8] Nallim L.G., Grossi R.O. (2007). Vibration of angle-ply symmetric laminated composite plates with edges elastically restrained. Compos. Struct..

[9] Secgin A., Sarigul A.S. (2008). Free vibration analysis of symmetrically laminated thin composite plates by using discrete singular convolution (DSC) approach: Algorithm and verification. J. Sound Vib..

[10] Cosentino E., Weaver P.M. (2010). An Enhanced Single-layer Variational Formulation for the Effect of Transverse Shear on Laminated Orthotropic Plates. Eur. J. Mech. A-Solids.

[11] Reissner E. (1945). The effect of transverse shear deformation on the bending of elastic plates. J. Appl. Mech..

[12] Mindlin R.D. (1951). Influence of rotary inertia and shear on flexural motions of isotropic elastic plates. J. Appl. Mech.-Trans. Asme.

[13] Whitney J.M. (1973). Shear Correction Factors for Orthotropic Laminates under Static Load. J. Appl. Mech..

[14] Bert C.W. (1973). Simplified Analysis of Static Shear Factors for Beams of NonHomogeneous Cross Section. J. Compos. Mater..

[15] Srinivas S., Rao C.V., Rao A.K. (1970). An exact analysis for vibration of simply-supported homogeneous and laminated thick rectangular plates. J. Sound Vib..

[16] Reddy J.N. (1984). A Simple Higher-Order Theory for Laminated Composite Plates. J. Appl. Mech..

[17] Reddy J.N. (1990). A general non-linear third-order theory of plates with moderate thickness. Int. J. Non-Linear Mech..

[18] Liew K.M., Huang Y.Q., Reddy J.N. (2003). Vibration analysis of symmetrically laminated plates based on FSDT using the moving least squares differential quadrature method. Comput. Meth. Appl. Mech. Eng..

[19] Karami G., Malekzadeh P., Mohebpour S.R. (2006). DQM free vibration analysis of moderately thick symmetric laminated plates with elastically restrained edges. Compos. Struct..

[20] Ngocong D., Maiduy N., Karunasena W., Tran-Cong T. (2011). Free vibration analysis of laminated composite plates based on FSDT using one-dimensional IRBFN method. Comput. Struct..

[21] Carrera E. (2007). On the use of transverse shear stress homogeneous and non-homogeneous conditions in third-order orthotropic plate theory. Compos. Struct..

[22] Chen C.C., Liew K.M., Lim C.W. (1997). Vibration analysis of symmetrically laminated thick rectangular plates using the higher-order theory and p-Ritz method. J. Acoust. Soc. Am..

[23] Aydogdu M. (2009). A new shear deformation theory for laminated composite plates. Compos. Struct..

[24] Ferreira A.J., Roque C.M., Martins P. (2004). Radial basis functions and higher-order shear deformation theories in the analysis of laminated composite beams and plates. Compos. Struct..

[25] Liu L., Chua L.P., Ghista D.N. (2007). Mesh-free radial basis function method for static, free vibration and buckling analysis of shear deformable composite laminates. Compos. Struct..

[26] Qu Y., Long X., Yuan G., Meng G. (2013). A unified formulation for vibration analysis of functionally graded shells of revolution with arbitrary boundary conditions. Compos. Part B-Eng..

[27] Murakami H. (1986). Laminated composite plate theory with improved in-plane responses. J. Appl. Mech..

[28] Sciuva M.D. (1986). Bending, vibration and buckling of simply supported thick multilayered orthotropic plates: An evaluation of a new displacement model. J. Sound Vib..

[29] Sciuva M.D. (1992). Multilayered anisotropic plate models with continuous interlaminar stresses. Compos. Struct..

[30] Kapuria S., Dumir P.C., Ahmed A. (2003). An efficient higher order zigzag theory for composite and sandwich beams subjected to thermal loading. Int. J. Solids Struct..

[31] Kapuria S., Achary G.G. (2004). An efficient higher order zigzag theory for laminated plates subjected to thermal loading. Int. J. Solids Struct..

[32] Kapuria S., Dumir P.C., Jain N.K. (2004). Assessment of zigzag theory for static loading, buckling, free and forced response of composite and sandwich beams. Compos. Struct..

[33] Kapuria S., Kulkarni S.D. (2007). An improved discrete Kirchhoff quadrilateral element based on third-order zigzag theory for static analysis of composite and sandwich plates. Int. J. Numer. Methods Eng..

[34] Pandit M.K., Sheikh A.H., Singh B.N. (2008). An improved higher order zigzag theory for the static analysis of laminated sandwich plate with soft core. Finite Elem. Anal. Des..

[35] Khandelwal R.P., Chakrabarti A., Bhargava P. (2012). An efficient FE model and Least Square Error method for accurate calculation of transverse shear stresses in composites and sandwich laminates. Compos. Part B-Eng..

[36] Carrera E. (2003). Historical review of Zig-Zag theories for multilayered plates and shells. Appl. Mech. Rev..

[37] Liew K.M., Hung K.C., Lim M.K. (1993). A continuum three-dimensional vibration analysis of thick rectangular plates. Int. J. Solids Struct..

[38] Vel S.S., Batra R.C. (2004). Three-dimensional exact solution for the vibration of functionally graded rectangular plates. J. Sound Vib..

[39] Zhou D., Cheung Y.K., Au F.T., Lo S. (2002). Three-dimensional vibration analysis of thick rectangular plates using Chebyshev polynomial and Ritz method. Int. J. Solids Struct..

[40] Zhou D., Au F.T., Cheung Y.K., Lo S. (2003). Three-dimensional vibration analysis of circular and annular plates via the Chebyshev-Ritz method. Int. J. Solids Struct..

[41] Zhou D., Lo S.H., Cheung Y.K., Au F. (2004). 3-D vibration analysis of generalized super elliptical plates using Chebyshev-Ritz method. Int. J. Solids Struct..

[42] Zhou D., Lo S.H., Au F.T., Cheung Y., Liu W. (2006). 3-D vibration analysis of skew thick plates using Chebyshev-Ritz method. Int. J. Mech. Sci..

[43] Zhou D., Cheung Y.K., Lo S.H., Au F. (2003). 3D vibration analysis of solid and hollow circular cylinders via Chebyshev-Ritz method. Comput. Methods Appl. Mech. Eng..

[44] Qu Y., Wu S., Li H., Meng G. (2015). Three-dimensional free and transient vibration analysis of composite laminated and sandwich rectangular parallelepipeds: Beams, plates and solids. Compos. Part B-Eng..

[45] Liew K.M., Kitipornchai S., Leung A.Y., Lim C.W. (2003). Analysis of the free vibration of rectangular plates with central cut-outs using the discrete Ritz method. Int. J. Mech. Sci..

[46] Kumar A., Shrivastava R.P. (2005). Free vibration of square laminates with delamination around a central cutout using HSDT. Compos. Struct..

[47] Sakiyama T., Huang M., Matsuda H., Morita C. (2003). Free vibration of orthotropic square plates with a square hole. J. Sound Vib..

[48] Gutierrez R.H., Laura P.A., Rossit C.A. (2000). Fundamental frequency of transverse vibration of a clamped rectangular orthotropic plate with a free-edge hole. J. Sound Vib..

[49] Larrondo H.A., Avalos D.R., Laura P.A., Rossi R. (2001). Vibrations of simply supported rectangular plates with varying thickness and same aspect ratio cutouts. J. Sound Vib..

[50] Avalos D.R., Laura P.A. (2003). Transverse vibrations of simply supported rectangular plates with two rectangular cutouts. J. Sound Vib..

[51] Laura P.A., Avalos D.R. (2008). Small amplitude, transverse vibrations of circular plates with an eccentric rectangular perforation elastically restrained against rotation and translation on both edges. J. Sound Vib..

[52] Shufrin I., Eisenberger M. (2016). Semi-analytical modeling of cutouts in rectangular plates with variable thickness–Free vibration analysis. Appl. Math. Modeling.

[53] Kwak M.K., Han S. (2007). Free vibration analysis of rectangular plate with a hole by means of independent coordinate coupling method. J. Sound Vib..

[54] Huang B., Wang J., Du J., Ma T., Guo Y., Qian Z. (2016). Vibration analysis of a specially orthotropic composite laminate with rectangular cutout using independent coordinate coupling method. Composite Structures.

[55] Guo Y., Jiang Y., Huang B. (2019). Independent coordinate coupling method for vibration analysis of a functionally graded carbon nanotube-reinforced plate with central hole. Adv. Mech. Eng..

[56] Du J.T., Li W.L., Liu Z.G., Xu H.A., Ji Z.L., Li W.L. (2011). Acoustic analysis of a rectangular cavity with general impedance boundary conditions. J. Acoust. Soc. Am..

[57] Sheikh A.H., Haldar S., Sengupta D. (2004). Free Flexural Vibration of Composite Plates in Different Situations Using a High Precision Triangular Element. J. Vib. Control.

